# Accuracy estimation of foamy virus genome copying

**DOI:** 10.1186/1742-4690-6-32

**Published:** 2009-04-06

**Authors:** Kathleen Gärtner, Tatiana Wiktorowicz, Jeonghae Park, Ayalew Mergia, Axel Rethwilm, Carsten Scheller

**Affiliations:** 1 Universität Würzburg, Institut für Virologie und Immunbiologie, Versbacher Str 7, 97078, Würzburg, Germany; 2 Department of Infectious Disease and Pathology, College of Veterinary Medicine, University of Florida, Gainesville, FL, USA

## Abstract

**Background:**

Foamy viruses (FVs) are the most genetically stable viruses of the retrovirus family. This is in contrast to the *in vitro *error rate found for recombinant FV reverse transcriptase (RT). To investigate the accuracy of FV genome copying *in vivo *we analyzed the occurrence of mutations in HEK 293T cell culture after a single round of reverse transcription using a replication-deficient vector system. Furthermore, the frequency of FV recombination by template switching (TS) and the cross-packaging ability of different FV strains were analyzed.

**Results:**

We initially sequenced 90,000 nucleotides and detected 39 mutations, corresponding to an *in vivo *error rate of approximately 4 × 10^-4 ^per site per replication cycle. Surprisingly, all mutations were transitions from G to A, suggesting that APOBEC3 activity is the driving force for the majority of mutations detected in our experimental system. In line with this, we detected a late but significant APOBEC3G and 3F mRNA by quantitative PCR in the cells. We then analyzed 170,000 additional nucleotides from experiments in which we co-transfected the APOBEC3-interfering foamy viral *bet *gene and observed a significant 50% drop in G to A mutations, indicating that APOBEC activity indeed contributes substantially to the foamy viral replication error rate *in vivo*. However, even in the presence of Bet, 35 out of 37 substitutions were G to A, suggesting that residual APOBEC activity accounted for most of the observed mutations. If we subtract these APOBEC-like mutations from the total number of mutations, we calculate a maximal intrinsic *in vivo *error rate of 1.1 × 10^-5 ^per site per replication. In addition to the point mutations, we detected one 49 bp deletion within the analyzed 260000 nucleotides.

Analysis of the recombination frequency of FV vector genomes revealed a 27% probability for a template switching (TS) event within a 1 kilobase (kb) region. This corresponds to a 98% probability that FVs undergo at least one additional TS event per replication cycle. We also show that a given FV particle is able to cross-transfer a heterologous FV genome, although at reduced efficiency than the homologous vector.

**Conclusion:**

Our results indicate that the copying of the FV genome is more accurate than previously thought. On the other hand recombination among FV genomes appears to be a frequent event.

## Background

Retroviral genomes are highly susceptible to the introduction of mutations, most of which are assumed to result from the action of the viral RT. While the contribution of the host RNA polymerase II to retroviral mutations has long been speculated [1], RNA polymerase II is now assumed to be a high fidelity polymerase because of its 3' to 5' repair activity [2,3]. Moreover, the significant variation in the *in vivo *mutation rates of different retroviruses suggests a host-independent source of mutations [4,5].

Retroviruses are pseudo-diploid and usually generate one DNA copy from the two RNA copies that are packaged into the viral particle. During reverse transcription, the RT enzyme can jump from one template strand to the other, thereby generating a hybrid transcript. If the two RNA templates are not identical, these template switching (TS) events can contribute to the overall retroviral mutation rate [6-10]. These TS events can occur even between distantly related retroviruses, provided that the different viruses can cross-package the heterologous viral genomes [11].

FVs, the only genus in the spumaretrovirus subfamily of *Retroviridae*, are known to be genetically extremely stable and have co-evolved with their host species [12-19] over more than 60 million years and represent the genetically most stable viruses which have an RNA phase in replication [12-19]. The biochemical and biological reasons for this stability have yet to be determined. It may be that the error rate of the FV RT is exceptionally low among retroviruses. However, the *in vitro *error rates of the human immunodeficiency virus (HIV) type 1 and of the prototype FV (PFV) RTs have recently been compared [20]. The overall probability of generating mutations was determined to be 7.5 × 10^-5 ^mutations per nucleotide per replication cycle for HIV-1 RT and 1.7 × 10^-4 ^for PFV RT [20]. Single nucleotide substitutions contributed to this with 6.3 × 10^-5 ^(HIV-1) and 5.8 × 10^-5 ^(PFV) mutations/nt per replication cycle. The remaining mutations were found to be due to insertions and deletions [20]. Thus, it appears that the rate of point-mutation during HIV-1 and PFV replication are remarkably similar, raising the possibility that a very low *in vivo *FV replication rate is the main reason for their genetic stability. Alternatively, the relatively high FV RT error rate that has been reported may be due to the specific *in vitro *assay conditions. This prompted us to analyze the *in vivo *mutation rate of PFV RT.

TS events happen frequently among plus-strand RNA-containing viruses and require the simultaneous infection of one cell by two parental viruses. Frequencies of TS have been studied for several retroviruses, particularly for HIV [7,21-26]. For instance, the development of resistance to antiviral therapy can be a consequence of recombination events during reverse transcription [27,28]. Through the use of retroviral vectors in single replication assays, the TS rates of HIV-1 and murine leukemia virus (MLV) were determined to be in the range of 3–4 crossovers per genome per replication cycle [26,29]. This is not necessarily reflected by the *in vivo* recombination rate that differs between HIV-1 and MLV [26,29]. Recombination rates were found experimentally to be 42.4% and 4.7% per 1 kb for HIV-1 and MLV, respectively [21,24].

Previous *in vitro *analysis has shown that the PFV RT creates a large number of small and large deletions, which suggest that the PFV RT jumps to an upstream site of the same strand during polymerization [20]. A high processivity of PFV RT [30] was proposed to be responsible for this slippage [20]. However, jumping to an upstream site *in vivo *may also result in a template switch. Since the *in vivo *TS rate of FVs has not thus far been determined, we have examined this mechanism using PFV in single replication assays. In addition, we investigated the ability of PFV particles to cross-transfer the genome of the related simian FV from macaque (SFVmac) and *vice versa *to estimate the probability and biological significance of TS in FVs. This may be particularly relevant in the light of recent findings on *trans*-species SFV infections of humans in non-occupational settings and in the case of HIV/FV double infections in humans [31-33].

Retroviral vectors are frequently used in gene transfer protocols and have been applied successfully in the clinical setting [34]. Considering an average clinical preparation of approximately 10^9 ^vector particles of a 10 kb vector, such a pharmaceutical product would harbor approximately 10^8 ^variants, assuming an RT point-mutation rate of 10^-5 ^per nucleotide per replication round. Thus, the accuracy of a retroviral RT enzyme and, furthermore, the chances to mobilize an integrated vector genome by superinfection with a homologous or heterologous virus appear to be critical factors to examine, especially since FV vectors are close to being used for clinical applications in humans [35-37].

## Results

### Analysis of FV mutation frequencies in the absence of Bet

**Figure 1:**
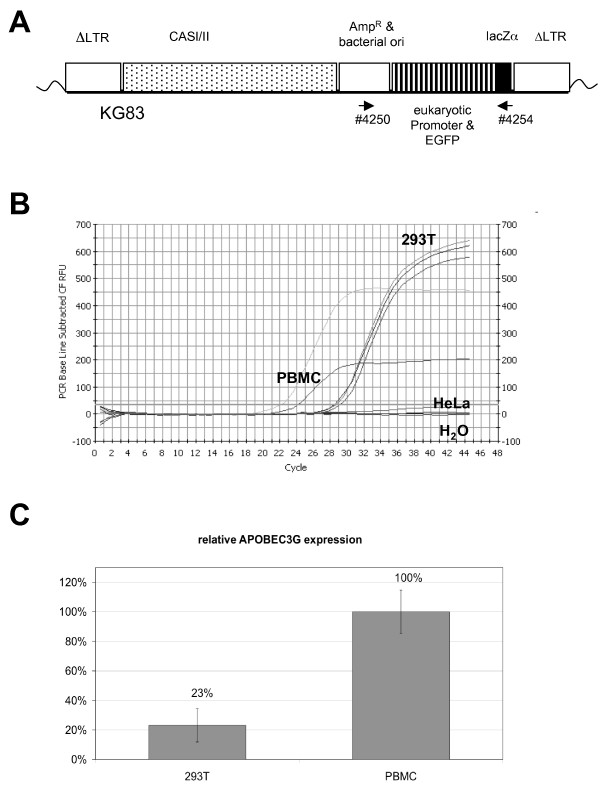
**Analysis of the FV *in vivo *mutation rate and APOBEC3G expression**. (A) Construct pKG83 used to evaluate the FV mutation rate *in vivo*. Marker gene EGFP was used for identification of infected cells. The locations of the primers (#4250 and #4254) used to amplify proviral sequences for sequencing are indicated. (B) Quantitative determination of APOBEC3G mRNA in HEK 293T (three runs) and HeLa cells as well as in PMBCs (two runs of two different PBMC preparations). H_2_O served as negative control. (C) Relative amounts of APOBEC3G mRNA in HEK 293T cells and in PBMCs (set to 100%) with respect to the amounts detected for the three housekeeping genes β-actin, GAPDH, and SDHA.

A previous analysis of the fidelity of the PFV RT used a recombinant enzyme and an assay that depended on the functionality of an indicator gene [20]. This raises the possibility that silent mutations could have been missed. To estimate the *in vivo *PFV RT mutation rate, we produced the replication deficient FV vector KG83 in HEK 293T cells (Fig. 1A). Following the transduction of HeLa recipient cells, we sorted single EGFP-positive cells into 96 well plates to obtain monoclonal cell cultures that each carries one FV provirus. The individual proviral sequences from a total of 346 clones were amplified by PCR using Pwo polymerase, and the resulting PCR products were sequenced. As PCR-introduced errors are randomly distributed over the transcript and the amplicon pool, it is unlikely that an individual PCR-introduced mutation will be detected in the sequencing reaction. However, in order to exclude such false positive events, we amplified and sequenced each mutation-carrying provirus twice. For a direct comparison of results we included the same genetic element in our analysis that has been used in the previous study by Boyer *et al*. [20].

Initially, we sequenced a total of 93,003 bases from 110 single cell-derived colonies and detected 39 point mutations, resulting in an error rate of approx. 4.2 × 10^-4 ^per base per replication cycle (Table 1).

**Table 1:** PFV point mutations identified after a single round of replication in the absence or presence of Bet.

	vector packaging in 293T cells w/o Bet	vector packaging in 293T cells with Bet
type of mutation	number of mutations in a total of 93,003 nucleotides	number of mutations in a total of 172,368 nucleotides

G→A	39	35

G→T	0	0

G→C	0	0

A→G	0	2

A→T	0	0

A→C	0	0

T→G	0	0

T→A	0	0

T→C	0	0

C→G	0	0

C→A	0	0

C→T	0	0

total	39	37

error rate	4.2 × 10^-4^	2.1 × 10^-4^

Vector particles were packaged in HEK 293T cells in the absence or presence of a bet expression plasmid. Vector preparations were used to transduce HeLa cells. Singly transduced cells were sorted into 96 well plates to form monoclonal cell cultures that carry a single provirus. Proviral DNA was amplified by PCR and sequenced in order to assess the *in vivo *error rate of FV replication.

### APOBEC3 expression in HEK 293T cells

All detected mutations were G to A transitions suggesting that APOBEC3 activity may be the driving force for mutations in our experimental system. This was surprising as previous studies documented the absence of APOBEC3G in HEK 293T cells by Western blotting [38]. Similarly, we could not detect APOBEC3G protein in these cells by Western blotting (data not shown). However, using the more sensitive quantitative RT-PCR for APOBEC3G, we measured a late (compared to PBMC) but significant PCR signal in these cells, whereas HeLa cells were negative for APOBEC message (Fig. 1B). APOBEC3G levels in 293T cells were about 25% of the amount detected in PBMC (Fig. 1C). APOBEC3F mRNA was even more abundant and was found to be almost four-fifths of the PBMC level (Additional file 1, Fig S3). To exclude the possibility that this feature was unique to the HEK 293T cells used in our laboratory, we also analyzed HEK 293T cells directly purchased from the American Type Culture Collection and detected the same signal. Thus, HEK 293T cells appear to express restriction factors that may influence the generation of foamy viral and other retroviral vectors produced in these cells.

### Analysis of FV mutation frequencies in the presence of Bet

In order to challenge the hypothesis that APOBEC activity is the driving force for the mutations in our experimental system, we analyzed 172,368 additional nucleotides from 236 individual cell clones from experiments in which we co-transfected 293T cells with the APOBEC3-inhibiting foamy viral *bet *gene. Bet has been demonstrated to inhibit APOBEC3G-triggered G-to-A mutations [38-40]. One group, however, has shown that Bet does not counteract APOBEC3G-mediated block of FV infectivity, as wildtype FV strains were similarly susceptible to APOBEC when compared with strains with a delta-bet mutation [41]. Introducing Bet into our model system, we observed a significant (χ^2 ^= 10.13) drop in mutations by 50%, indicating that APOBEC activity indeed contributed substantially to the foamy viral replication error rate in HEK 293 T cells (Table 1). Bet co-transfection reduced G to A exchanges not only in the GG context that was reported to be a preferential target site for APOBEC3G in the HIV genome [42], but also in other sequence contexts (Table 2). In line with this, a study published by Delebecque and colleagues did not identify such GG hotspots for APOBEC3G-mutagenesis in the foamy viral genome [41].

**Table 2:** Sequence context of G to A mutations in the absence or presence of Bet.

	vector packaging in 293T cells w/o Bet	vector packaging in 293T cells with Bet
G to A mutation with sequence context	number of mutations in a total of 93,003 nucleotides	number of mutations in a total of 172,368 nucleotides

**G**G→**A**G	8	10

**G**A→**A**A	15	16

**G**T→**A**T	2	1

**G**C→**A**C	14	8

G to A mutations from Table 1 categorized according to their sequence context.

As the total amount of residual APOBEC activity in our bet-cotransfection experiments is unclear, we cannot exactly pin down the APOBEC-independent error rate of foamy viral replication. However, it seems very unlikely that the majority of the G to A mutations in the presence of Bet is caused by an intrinsic activity of FV RT as the study of Boyer *et al. *found that only 1 out of 3 nucleotide substitutions caused by FV RT is a G to A exchange [20]. As we detected only 2 non-G-to-A mutations within the total of 265,371 sequenced nucleotides in our study, one would expect to find no more than one additional G to A transition intrinsically caused by foamy viral RT. In this light, it seems very likely to us that the remaining G to A mutations that we detected in the presence of Bet were caused by residual APOBEC enzyme activity. This scenario results in a corrected error rate of 3 mutations in 265,371 nucleotides (i.e. an error rate of 1.1 × 10^-5 ^per site per replication).

Within the 265,371 nucleotides that we sequenced, we identified only a single deletion (which occurred in the experiments with Bet expression) and no insertion (see also Additional file 1, Fig. S4). This is in sharp contrast to the situation reported with recombinant FV RT where such mutations accounted for the majority of all mutations identified [20]. This probably indicates differences between the *in vitro *and the *in vivo *accuracy of FV reverse transcription. The one 49 bp deletion we observed took place at a DNA stretch with no obviously repeated sequence (Additional file 1, Fig. S4). Boyer *et al*. also reported that deletions occurring during FV reverse transcription do not necessarily involve repeated sequences [20].

### Experimental design to determine FV TS

We determined the TS rate of FV by a phenotypic resistance assay described by Anderson *et al*. for MLV [21]. As an internal quality control for the assay, we similarly determined the MLV TS rate and found it to be in the same range (7.1% ± 2.0% SE for a 1 kb fragment, data not shown) as what has been reported previously (4.7%, [21]).

**Figure 2:**
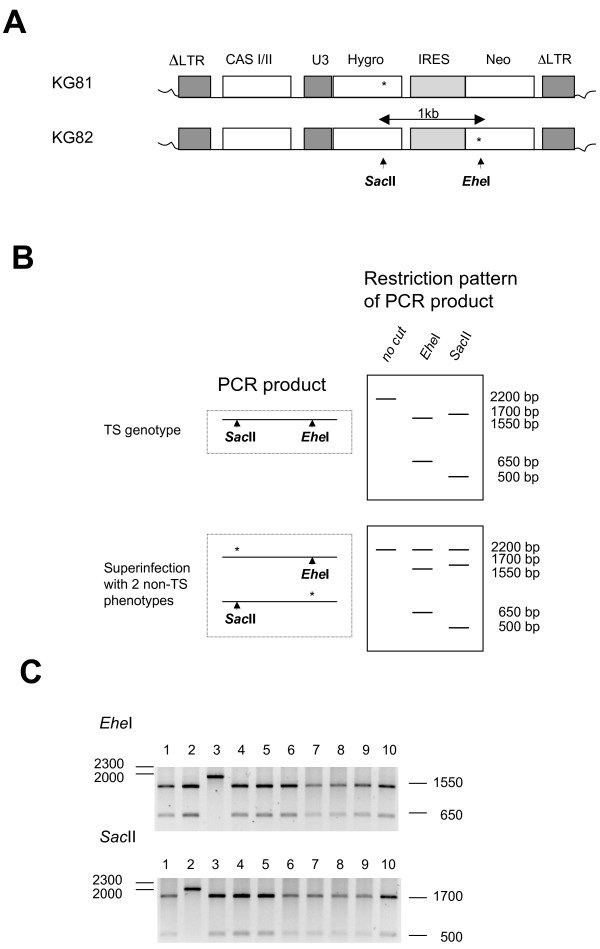
**Template Switching (TS) rate of foamy viral replication**. (A) MD9-derived PFV vector viruses (KG81 and KG82) expressing hygromycin and neomycin resistance genes under control of a SFFV U3 promotor. CASI/II are *cis*-acting sequences required for FV vector transfer [71]. KG81 carries a point mutation in the hygromycin resistance gene that abolishes its function and destroys a SacII restriction site. KG812 carries a point mutation in the neomycin resistance gene that abolishes its function and destroys an EheII restriction site. The two mutations are 1 kb apart. (B) Distinction of TS events from superinfection with KG81 and KG82 by restriction pattern: amplification of the proviral sequences by PCR generates a 2.2 kb fragment with a SacI site at position 500 and an EheII site at position 1550. Amplicons of TS events carry the two intact restriction sites and show a restriction pattern depicted in the upper box, whereas amplicons of superinfected cells show the restriction pattern depicted in the lower box. (C) Representative digests of 10 clones from the TS experiment. All 10 clones show the expected pattern for TS events. Upper lane 3 and lower lane 2 show incomplete digests.

For the determination of the foamy viral TS rate, we constructed the vectors KG81 and KG82 that carry the resistance genes for hygromycin and neomycin (Fig. 2A). KG81 carries a mutation in the Hygro resistance gene that destroys both the resistance activity as well as a pre-existing *Sac*II restriction site. KG82 has a mutation in the Neo resistance gene that destroys both the resistance activity as well as a preexisting *Ehe*I site (the function of the restriction sites will be discussed later). Both mutations are 1 kb apart.

Vector production was performed by transfection of 293T cells with a mixture of KG81 and KG82 together with gag-pol-env-helper plasmids. The resulting vector preparation consisted of virions that contained either the original KG81 or KG82 sequences or KG81/KG82 hybrid sequences as a result of template switching. This vector preparation was used to transduce HEK 293 cells that were grown in the presence of neomycin and/or hygromycin. The resulting colonies were quantified.

Colonies carrying the KG81 provirus are resistant to neomycin (Neo^r^) but sensitive to hygromycin (Hygro^s^). Colonies carrying the KG82 provirus are sensitive to neomycin (Neo^s^) but resistant to hygromycin (Hygro^r^). These phenotypes will be referred to as non-TS phenotypes. A double-resistant colony (Neo^r ^Hygro^r^) carries a provirus that is the result of a TS event within the 1 kb region between the mutation sites, a phenotype that will be referred to as TS-phenotype.

The TS rate in this 1 kb region can be calculated from the ratio of TS-phenotype colonies versus the total number (TS and non-TS phenotypes) of colonies. If no TS occurred, vectors displayed either the phenotype Neo^s ^and Hygro^r ^or Neo^r ^and Hygro^s^. In the case of a TS event, vectors were either Neo^r ^plus Hygro^r ^or Neo^s ^plus Hygro^s^. The number of TS events can be quantified on culture plates supplemented with the two antibiotics, allowing the outgrowth of one of the two TS phenotypes (Neo^r ^plus Hygro^r^). Since the other TS phenotype (Neo^s ^plus Hygro^s^) will be suppressed, the number of TS events is twice as high as the number of colonies on the double antibiotics plate. On plates supplemented with only one of the two antibiotics, the respective non-TS phenotypes will grow out as well as the double resistant TS-phenotype. The number of non-TS colonies on these plates can therefore be calculated by subtracting the number of double resistant colonies (counted from the double antibiotics plate) from the total number of colonies visible on each single antibiotic plate.

Double resistant colonies can not only result from the transduction with TS-genotypes but can also result from a superinfection with both KG81 and KG82. (To minimize superinfection we transduced the cells with an m.o.i. < 0.01). Superinfections can easily be distinguished from transductions with TS-genotypes by amplification of the proviral DNA and subsequent digestion with *SacII *and *EheI*; whereas the PCR product of a TS genotype carries both the intact *SacII *and the intact *EheI *site on a single molecule (and will therefore generate a positive restriction pattern with the two enzymes); the PCR products from superinfected cells carry the restriction sites on different molecules (and will therefore produce a different restriction pattern). The digestion of the 2.2 kb amplicons with *Ehe*I and *Sac*II should result in bands of 1.55 and 0.65 kb or 1.7 and 0.5 kb respectively if a recombination event had occurred between the two sites 1 kb apart (Fig. 2B). If double resistance was caused by superinfection with two viruses, the digestions would show a third band. This latter band would have been caused by the uncut 2.2 kb amplicon of one provirus (Fig. 2B).

### Analysis of the FV TS rate

Table 3 summarizes the values obtained for the FV TS rate in the transient assay. Within the investigated 1 kb region we calculated an average recombination rate of 22.3% ± 0.27% SE from the results of three independent experiments. When the proviruses of 26 cell colonies were analyzed by PCR and restriction enzyme digestion for the presence of superinfections with two vectors, we exclusively found evidence for recombinant proviruses, demonstrating that the calculated TS rate is not biased by false-positive colonies. A representative digestion pattern is shown in Fig. 2C.

**Table 3:** Calculation of the PFV TS rate.

	Column 1	Column 2	Column 3	Column 4	Column 5	Column 6	Column 7
Selection Medium	Neo + Hygro	Neo	Hygro	**TS Phenotype**	**NON-TS Phenotype (Neo)**	**NON-TS Phenotype (Hygro)**	TS rate per 1 kb

Experiment 1	3,200	7,000	21,000	**6,400**	**3,800**	**17,800**	**22.9%**

Experiment 2	4,300	16,500	22,500	**8,600**	**12,200**	**18,200**	**22.1%**

Experiment 3	600	4,100	1,400	**1,200**	**3,500**	**800**	**21.8%**

average							**22.3%**

KG81 and KG82 (Fig. 2A) were combined and packaged in 293T cells. Recipient HEK 293 cells were transduced with vector preparations and cells were grown on culture plates supplemented with either neomycin, hygromycin, or both. Colonies were quantified. Column1: number of colonies counted on plates with Neomycin and Hygromycin; column 2: number of colonies counted on plates with Neomycin; column 3: number of colonies counted on plates with Hygromycin; column 4: TS phenotypes (including the double sensitive phenotype that does not grow on the double antibiotics plate) calculated as 2× column 1 (e.g. 2 × 3,200 = 6,400); column 5: Non-TS phenotypes (Neo^R^, Hygro^S^) calculated as column 2 - column 1 (e.g. 7,000 - 3,200 = 3,800); column 6: Non-TS phenotypes (Neo^S^, Hygro^R^) calculated as column 3 - column 1 (e.g. 21,000 - 3,200 = 17,800); column 7: TS rate within 1 kb fragment calculated as 100×(column 4/[column 4 + column 5 + column 6]) (e.g. 100 × (6400/[6400+3800+1780]) = 22.9%).

As the TS rate is in the range of 20%, one would assume that one out of 5 TS-viruses would undergo a second TS event, resulting again in a mixed resistant phenotype (Neo^s ^and Hygro^r ^or Neo^r ^and Hygro^s^) that would not show up on the double antibiotics plate. The real, partly hidden TS rate is therefore about 20% higher than the observable TS rate, so that the true PFV TS rate is probably in the range of 27% within the 1 kb region. For a typical FV RNA (pre-) genome of 12 kb, this would correspond to a 98% probability (calculated as P = 1-[1-0.27]^12^) to undergo at least one internal TS event per replication cycle.

Recombination may not be relevant to the application of FV-derived vectors, as both templates are identical in sequence, but it is clearly relevant for the generation of new viruses or new viral variants [14,27,28]. The recent analyses of SFV sequences from wild chimpanzees demonstrated the presence of frequent FV recombinants and the infection of chimpanzees by FVs from lower monkeys [14,43]. We therefore tried to estimate the probability of generating new FV recombinants by packaging heterologous viral sequences.

### Investigation of the transfer of FV vector genomes

**Figure 3:**
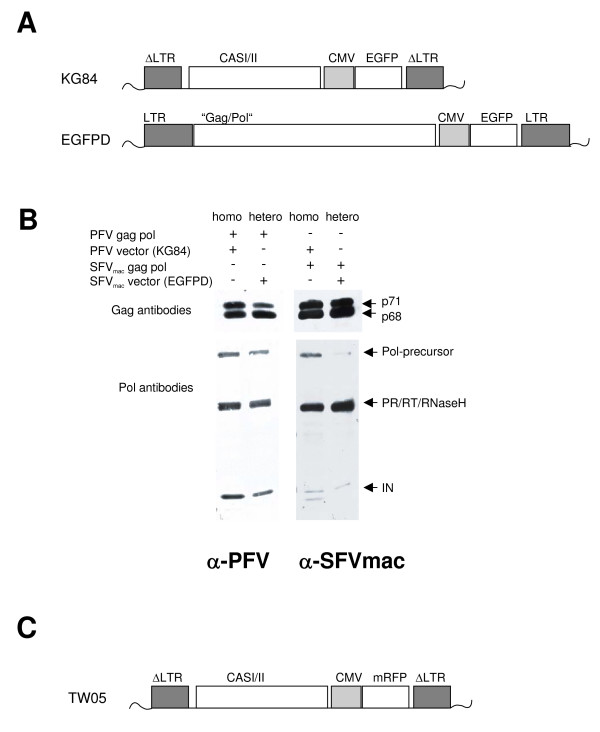
**Analysis of cross packaging**. **(A) KG84 (PFV) and EGFPD (SFVmac) vector viruses used in this analysis**. "Gag/Pol" of the EGFPD vector corresponds to the CASI/II region found in KG84. Due to point mutations of start and internal ATGs of the *gag *and *pol *ORFs no viral proteins are translated from the EGFPD vector virus. (B) Gag and Pol protein composition of PFV vector (KG84) and SFVmac vector (EGFPD) particles produced in the presence of homologous (homo) and heterologous (hetero) gag pol proteins. (C) Structure of the TW05 (PFV) vector virus expressing mRFP used to analyze the simultaneous transfer of PFV and SFVmac FV genomes.

To analyze the transfer of FV vector genomes, we investigated whether a given FV capsid is able to package and transfer a related, but clearly different FV genome. We used the vectors KG84 and EGFPD that were derived from PFV (from chimpanzee isolate) and SFVmac (from Asian monkeys) [44], respectively (Fig. 3A). To analyze the cross packaging activity of the two viruses, we packed the PFV vector with Gag Pol proteins derived from SFVmac and the SFVmac vector with Gag Pol proteins derived from the PFV vector. As an internal control, each vector was also packaged with its homologous Gag Pol proteins. There is evidence that (pre-) genomic RNA may have a structure-forming capability in FV particle assembly [45,46]. To exclude that this feature inhibits proper particle assembly in a cross packaging situation, we determined the protein composition of the vector particles being released into the supernatant by the packaging cells. As shown in Fig. 3B, we did not detect significant differences in the Gag and Pol protein composition between homologous and heterologous vector particles.

In order to determine the infectivity of the produced vectors, we transduced HT1080 fibroblastoid recipient cells with the supernatants from the different packaging cultures and determined the amount of EGFP-positive cells by flow cytometry. Table 4 summarizes the data obtained from the cross-packaging experiments. The results show that both PFV and SFVmac can be effectively cross-packed by heterologous Gag Pol proteins, although at a slightly lower efficiency.

**Table 4:** Transfer rates of PFV vector (KG84) and SFV_mac _vector (EGFPD) viruses on HT1080 fibroblastoid cells after cross-packaging with homologous and heterologous FV gag pol proteins.

**vectors and packaging systems**	**EGFP-positive target cells**	**Cross-packaging efficiency**
PFV gag pol +	51,3%	---
PFV vector		

PFV gag pol +	13,5%	26,4%
SFV_mac _vector		

SFV_mac _gag pol +	19,3%	---
SFV_mac _vector		

SFV_mac _gag pol +	6,4%	33,3%
PFV vector		

Vectors were packaged together with the indicated packaging proteins in 293T cells. Supernatants from packaging cells were used for transduction of HT1080 cells. Transduction rates were measured by detection of EGFP by flow cytometry. The cross-packaging efficiency is calculated from the ratio of EGFP-positive cells of the heterologous system versus the homologous system (e.g. 26.4% = 13.5%/51.3%).

In contrast to the experimental setup described above, the *in vivo *situation in which cross-packaging could occur – a co-infection of a cell with two different viruses – would represent an environment in which two viral genomes would compete to be packaged by one viral capsid. In order to determine the cross-packaging activity in the presence of the competing homologous system, we constructed another PFV vector (TW05) that expresses mRFP instead of EGFP (Fig. 3C) so that the PFV vector can be easily distinguished from the SFVmac vector EGFPD that encodes for EGFP. We then co-transfected HEK 293T cells with both vectors and packaged them with either PFV or SFVmac helper plasmids. Vector production was quantified by transduction of HT1080 cells and flow cytometric analysis of EGFP and mRFP expression. As depicted in Table 5, each of the two vectors was packaged with high efficiency by its homologous Gag Pol proteins in the presence of the competing heterologous vector. More importantly, however, both packaging systems allowed for the simultaneous packaging of the heterologous vector with a relative efficiency of 1.3% (SFVmac vector plus PFV particles) and 15% (PFV vector plus SFV particles).

**Table 5:** Competitive transfer rates of PFV (TW05, ref fluorescence) and SFV_mac _(EGFPD, green fluorescence) vectors following simultaneous packaging into PFV or SFV_mac _particles.

**vectors and packaging systems**	**fluorescence-positive cells**	**competitive cross-packaging efficiency**
PFV vector (red) +	68% red cells	---
PFV gag pol		

SFV_mac _vector (green) +	67% green cells	---
SFV_mac _gag pol		

PFV vector (red) +	63% red cells	---
SFV_mac _vector (green) +	0,85% green cells	1.3%
PFV gag pol		

PFV vector (red) +	6,8% green cells	---
SFV_mac _gag pol +	1,2% red cells	15%
SFV_mac _vector (green)		

Vectors were packaged together with the indicated packaging proteins in 293T cells. Supernatants from packaging cells were used for transduction of HT1080 cells. Transduction rates were measured by detection of EGFP and mRFP expression by flow cytometry. The competitive cross-packaging efficiency is calculated from the ratio of fluorescence-positive cells of the heterologous system versus the homologous + heterologous system (e.g. 1.3% = 0.85%/(63% + 0.85%)).

These results show that FV particles are in principle able to transfer heterologous but related sequences, albeit at a considerably lower efficiency in relation to the homologous vector genome. Furthermore, the transfer of heterologous genomes may not be reciprocal between different FVs.

## Discussion

FVs appear to be an exception to the majority of retroviruses in respect to their genome conservation. This virus is genetically very stable and, with the exception of *trans*-species transmissions, has co-evolved with its hosts [17]. Their high genome conservation often allows the designation to a particular monkey or ape subspecies through the analysis of the appropriate FV sequence [14,19]. Furthermore, in *trans*-species transmissions to humans or apes the transmitted virus can be easily traced back to the transmitting monkey species and appears to be genetically stable in the new host for decades [16,47,48].

We have demonstrated that G to A transitions dominate the error rate in foamy viral vector production in HEK 293T cells and that the *bet *gene has a substantial influence on the overall FV mutation rate *in vivo *as its presence reduced the number of mutations in our assay system by 50%. Our experimental data suggest that members of the APOBEC family rather than an intrinsic activity of FV RT are responsible for this mutation hotspot, so that the error rate of foamy viral replication would be in the range of 1.1 × 10^-5^.

This contrasts to what has been published previously for a recombinant assay system in which an overall error rate of 1.7 × 10^-4 ^has been determined. In this case, the error rate was dominated by deletions and insertions whereas nucleotide substitutions contributed to the error rate only with a factor of 5.8 × 10^-5 ^[20]. Within the 265,371 nucleotides that we sequenced we identified only a single deletion and no insertion (see also Additional file 1, Fig. S4). This probably indicates differences between the *in vitro *and the *in vivo *accuracy of FV reverse transcription. The one 49 bp deletion we observed took place at a DNA stretch with no obviously repeated sequence (Additional file 1, Fig. S4). Boyer *et al*. also reported that deletions occurring during FV reverse transcription do not necessarily involve repeated sequences [20]. Our results suggest a much higher *in vivo *accuracy of FV genome replication than has previously been thought. These findings are reminiscent of previous studies comparing the *in vivo *and *in vitro *mutation rates of HIV-1 [49,50].

Although this low mutation rate would be more consistent with FV genome conservation, it does not completely explain the genomic stability of FVs. For instance, even the extrapolated FV point-mutation rate is slightly higher than the point-mutation rate reported for primate T-lymphotropic virus type I (PTLV-I) [4]. However, FV evolved approximately ten times slower than PTLV-I in the living host, implying less *in vivo *genetic stability in the latter [17,51]. Thus, additional factors probably contribute to FV genome conservation.

The findings of this study suggest that the HEK 293T cell line, which is widely used in laboratories, exerts APOBEC activity that easily overrides the intrinsic error rate of foamy viruses. Our data suggest that APOBEC3G could be involved in the observed G-to-A hypermutating activity, although other members of the APOBEC family may contribute to this phenomenon as well. Russell *et al. *have published that foamy viral replication is vulnerable to a broad variety of APOBEC isoforms, including hA3A, hA3C, hA3F, and hA3G [52]. It is beyond the scope of this study to identify which of these isoforms are expressed in HEK 293T cells, but a further characterization of this cell line in this regard remains a desirable task.

To reverse transcribe the virus RNA, the retroviral RT must naturally perform the switches of templates either intra- or inter-molecularly. Therefore, the aspect of TS is not easy to accurately analyze. Furthermore, different conditions exist that have major influences on the recombination rate by TS, such as the role of nucleocapsid (NC) protein [53,54], a preferential homodimer packaging of the genomic RNA [55-57], the accessibility to the recombination machinery [29], the possible presence of recombination hotspots [58], properties of the RT enzyme complex [29], and the overall homology of the two packaged genomes. On the other hand, HIV-1 and -2, which make use of different mechanisms of genome packaging [59], recombine at similar rates [22]. Recombination between these two viruses which have a relatively low overall homology has also been reported [11]. FVs do not have a NC protein [60,61] and all the other points, in particular whether homo- or hetero-dimers are preferentially packaged, are still unknown. In addition, their replication pathway diverges from the orthoretroviral strategy [60,61]. However, they package two copies of (pre-) genomic RNA [62,63], and therefore are theoretically suited to recombine by TS. Recombination of FV genomes has been shown recently for wild chimpanzee FVs [14] and superinfection by SFVs from lower monkeys to chimpanzees has been documented [14,43]. To get a first insight into the frequency of FV TS we analyzed this by methods which have been established to measure MLV recombination [21]. Our results indicate that recombination of FVs by TS occurs at a higher frequency than MLV [21], but at a lower frequency than HIV-1 [24]. Clearly, additional studies will be required to more precisely determine the FV TS rate.

An issue which becomes relevant for recombination by TS is the packaging of two different genomes in one capsid. Thus, we investigated the probability of cross-vector transfer of two related but clearly different FVs in the presence or absence of the homologous genome. The ability to cross-package a heterologous retroviral genome has been extensively investigated among lentiviruses, with the likelihood of cross-packaging increasing with the relatedness of the viruses in question. However, a non-reciprocal packaging was found when for instance HIV-1 and -2 were investigated [64-68]. Our results show that in the FV system, heterologous vector transfer is possible although less efficient than the homologous transfer. This implies the packaging of heterologous (pre-) genomic RNAs and may have consequences for the possibility of generating new FVs by recombination. Given the differences in spuma- and ortho-retroviral replication pathways in general [60,61], and especially in the packaging, assembly, and conditions of reverse transcription [61], a recombination by TS requiring co-packaging of spuma- and ortho-retroviral genomes appears unlikely. However, we did not investigate this issue, and it may be worth doing so in future experiments.

Although heterologous vector transfer was reduced, particle composition was found to be unaltered. In particular, the cleavage of the Gag precursor proteins indicative of Pol protein incorporation was found to be unaffected by the type of vector genome packaged. It is known that Pol encapsidation appears to somehow depend on the presence of viral RNA which is packaged by a still unknown mechanism [45,46]. Our results show that we may have established an experimental system that will allow this to be more accurately defined.

In terms of genome conservation, our results show that FVs undergo recombination by TS during reverse transcription relatively easily, but they have a much lower mutation rate than previously thought. This is clearly the case for deletions that occurred only at a very low frequency in the *in vivo *assay but probably also pertain to the frequency of point-mutations. In order to avoid alterations to their genome, FVs appear to have developed a particular strategy through the generation of the Bet protein. This may help us to better understand the conundrum of error prone reverse transcription and FV genome conservation.

## Methods

### Recombinant DNA

Standard techniques in molecular biology [69,70] were used to generate the plasmids described below. Vector viruses were abbreviated with the plasmid name lacking the 'p'. Sequence data of oligonucleotide primers are available as supporting online material (Additional file 1, Fig. S1 at ).

(i) The pKG83 vector, used to determine the FV point-mutation rate, is shown in Fig. 1A. In a pMD9-derived backbone it contains [71] within the PFV internally-deleted LTRs (lack of U3 region), the cassette with the bacterial origin of replication, the ampicillin resistance gene, a eukaryotic SFFV-derived promoter which directs gene expression of EGFP, and the α-fragment of the *lac*Z gene. It was constructed by generating the intermediate vector pMH119 from pMH118 [72] by ligating the pLIB-(Clontech) derived *Bsm*BI/blunt 1.65 kb PCR product (amplified with primers #4300/#4301 and representing the bacterial origin of replication and the ampicillin resistance gene) with the 6.78 kb pMH118 *Bsm*BI/*Sal*I and the 4.59 kb pMH118 *Sal*I/blunt fragments in a three-fragment ligation. Exchange of the gag/pol part of pMH119 for the *cis*-acting sequences (CAS I/II) of pMD9 as a 2.6 kb *Eco*RI/*Mlu*I fragment and insertion of a pUC19-derived PCR cassette as a 0.45 kb fragment (amplified with primers #4229/#4230 and representing the α-fragment of the bacterial *lac*Z gene) into the single *Not*I site of this vector generated pKG83. Transfer of pKG83 allowed the sorting of transduced eukaryotic cells via their green fluorescence and the sequencing of the bacterial parts, which were not under selective pressure in eukaryotes.

(ii) To create the PFV vectors pKG80, pKG81, and pKG82, the Hygro-IRES-Neo gene cassettes from the parental plasmids pJS30, pJA31-1 kb, and pJA32-1 kb [21] were amplified with the primers #1854/#1856 in the presence of 5% dimethylsulfoxide (DMSO), due to very high G-C content of the target DNA. The amplicons were inserted into the pCR2.1-Topo vector (Invitrogen). Following DNA sequencing the resistance gene cassettes were excised by digestion with *Bst*XI and *Eco*RV. Subsequently they were blunt end-inserted downstream of the internal SFFV U3 promoter into the pMD9 vector [71] that was digested with *Bam*HI and *Not*I to release the gene coding for EGFP. This resulted in plasmids pKG80, pKG81, and pKG82, which were completely sequenced in the amplified parts to exclude any unwanted nucleotide changes.

(iii) The SFVmac vector (pEGFPD) and packaging plasmids (pCIgag-1, pCIpol, and pCIenv3.5) will be described in detail elsewhere. In brief, the complete open reading frames (ORFs) of SFVmac gag, pol and env were inserted separately downstream of the chimeric β-globin-IgG intron of the pCI expression vector (Promega). In the SFVmac vector pEGFPD the ATG initiation codons of the *gag *and *pol *ORFs were inactivated by *in vitro *mutagenesis to TAG and additional stop codons were introduced into the *gag *ORF to abrogate translation from internal ATGs. Substitution of the SFFV U3 promoter in pMD9 [71] by the human cytomegalovirus (CMV) immediate early gene enhancer/promoter of pEGFPD generated pKG84. To create this, the CMV/EGFP expression cassette was released from pEGFPD as a 1.56 kb *Kpn*I/*Not*I fragment and blunt end inserted into pMD9 from which the SFFV U3/EGFP expression cassette had been excised as a 1.2 kb *Not*I/*Eco47*III fragment. The PFV packaging plasmids pCZIgag-2, pCZIpol, and pCZenvEM002 were described previously [71,73].

(iv) The pTW05 vector was made by amplifying the mRFP gene with primers #4404 and #4405 from plasmid 11935 mRFP-Ub (Addgene). Following sequence determination the resulting 0.67 kb fragment, digested with *Age*I/*Not*I, was inserted behind the CMV promoter of pKG84 in place of the EGFP gene which had been excised as a 0.73 kb fragment using the same restriction enzymes.

### Determination of FV in vivo mutation rate

2 × 10^6 ^HEK 293T cells [74], seeded in a 6 cm dish, were transfected with a total amount of 6 μg DNA (1.5 μg of pKG83 vector and the three packaging plasmids pCZIgag-2, pCZIpol, and pCZenvEM002 [71,73] or with 1.5 μg vector and the packaging plasmids plus the 1.5 μg pLENbet construct [75]) using a polyethylenimine (PEI) protocol [73]. Following enhancement of viral transcription by the addition of 10 mM sodium butyrate for 8 hrs at 1 day post-transfection (d.p.t), the vector-containing supernatant was harvested after 48 hrs. After filtration through a 450 nm filter (Schleicher & Schuell) it was aliquoted and stored at -80°C. HeLa recipient cells were transduced with an efficiency below 5% [as determined by EGFP-detection in fluorescence-activated cell sorting (FACS)] to avoid superinfection of cells. Positive-scoring cells were sorted on a FACSDiVa (Beckton Dickinson) into 96 well plates at a rate of 1 EGFP-positive cell per well to allow the generation of monoclonal cell cultures. Cells were grown to confluence, and transferred to 12 well plates. Total DNA was harvested with the Qiagen Blood and Tissue kit and subjected to a PCR reaction with primers #4250 and #4254 and Pwo polymerase (PeqLab). The resulting 2.2 kb amplicon was purified with the GenElute PCR Clean-Up kit (Sigma) and the prokaryotic parts were directly sequenced with BigDye Terminator v1.1 on an ABI PRISM 3100 Genetic Analyzer (Applied Biosystems) using primers #4257 or #4265. A total of 110 cell colonies from KG83 vector-transduced cells in the absence of Bet protein and 236 colonies from cells transduced with KG83 vector and transient Bet protein-expression were analyzed this way.

### Quantitative RT-PCR

Total cellular RNA was harvested from 5 × 10^6 ^HEK 293T, HeLa, and PBMCs, obtained from the Department of Transfusion Medicine (Universität Würzburg). RNA was extracted using the RNeasy kit (Qiagen) following the manufacturers instructions. After elution in 50 μl RNase-free water, 1 μg of RNA was used for cDNA synthesis using the iScript cDNA Synthesis kit (BioRad) that primes the cDNA synthesis with oligo(dT). Real time PCR was performed on an iCycleriQ Multicolor Real-Time PCR detection system (BioRad) utilizing 1 μl of the cDNA reaction, SYBRGreen as fluorophore, and primer pairs able to amplify transcripts of APOBEC3G (#4302) and 3F (#4286/#4287), β-actin (#4304), GAPDH (#4305), and the A subunit of SDHA (#4303). The levels of APOBEC mRNA in the individual cells were normalized to the levels of the housekeeping genes (β-actin, GAPDH, A subunit of SDHA) using the geNorm VBA Applet for Excel [76]. All assays were independently carried out at least three times.

### TS assay

Vector virus-containing supernatants were obtained by transfection of 2 × 10^6 ^HEK 293T cells, seeded in 6 cm dishes, with a total amount of 6 μg DNA (0.75 μg KG81, 0.75 μg KG82, and 1.5 μg of each of the three gag, pol, env helper plasmids) using PEI [73]. Transcription was enhanced by the addition of 10 mM sodium butyrate for 8 hrs at 1 d.p.t. The supernatants were harvested 2 d.p.t, passed through a 450 nm filter (Schleicher & Schuell), and stored at -80°C. The vector titers were determined on HEK 293 target cells by single selection with 600 μg/ml G418 (Roth) or 250 μg/ml Hygro (Roth) and double selection with 400 μg/ml G418 plus 150 μg/ml Hygro. The TS rate was calculated by the equation: TS rate = number of TS phenotypes/(number of TS phenotypes + number of non-TS Phenotypes). For production of single cell colonies, HEK 293 target cells were transduced at a multiplicity of infection (MOI) below 0.01 to avoid double infections and selected for two weeks with medium containing the antibiotic concentrations as described. To characterize recombinant proviruses, we amplified the proviral vector genomes from double resistant colonies by a nested PCR using the first round primers #1854/#4294 and the second round primers #1855/#1857. One sixth of the reaction from the first PCR cycle was employed in the nested reaction. Both amplification cycles were performed in the presence of 5% DMSO due to very high G-C content of the target DNA. The amplicons were analyzed by agarose gel electrophoresis following digestion with *Ehe*I and *Sac*II.

### Vector transfer assays

6 × 10^6 ^HEK 293T cells seeded in 10 cm dishes, were transfected with a total amount of 16 μg DNA (4 μg of the PFV vector pKG84 or the SFVmac vector pEGFPD and 4 μg of each of the three packaging constructs for PFV or SFVmac) using a PEI protocol [73]. After 48 hrs, HT1080 fibroblastoid target cells were exposed to a 1:100 dilution of 0.45 μm filtered supernatant (Schleicher & Schuell) and analyzed two days later for vector transfer by flow cytometry with FACSCalibur (Beckton&Dickinson) using the Cell Quest Pro software. The normalized cross-transfer rate was calculated as the percentage of SFVmac vector transfer packaged with PFV constructs in relation to the authentic PFV vector transfer and *vice versa*.

For the investigation of the simultaneous packaging of two FV genomes 2 × 10^6 ^HEK 293T cells seeded in a 6 cm dish, were transfected with 2 μg of each vector (pTW05 and/or pEGFPD) as well as 2 μg of each packaging plasmid using a PEI protocol. The total amount of DNA in the transfection mix was adjusted to 20 μg using pcDNA3.1zeo(+) plasmid (Invitrogen). The empty vector was also used to adjust for DNA differences resulting from omitting one vector. Following enhancement of transcription by the addition of 10 mM sodium butyrate for 8 hrs at 1 d.p.t the supernatant was harvested at 2 d.p.t, passed through a 0.45 μm filter (Millipore), diluted 1:10, and applied to 1.5 × 10^4 ^HT1080 fibroblastoid target cells. These were analyzed for EGFP and/or mRFP expression by FACS two days after transduction. Vector transfer assays were performed at least three times using different plasmid preparations.

### Immunoblotting

Lysis of HEK 293T cells was performed with lysis buffer in 6 cm dishes. Extracellular virions were prepared from the filtered supernatant of transfected cell cultures by ultracentrifugation through a 20% sucrose cushion in a Sorvall Surespin 630 rotor at 25,000 rpm, 4°C for 3 hrs. Viral particles and cellular lysates were resolved in protein sample buffer, separated in sodium dodecylsulfate-containing protein gels, blotted to nitrocellulose, and reacted with PFV Gag and Pol monoclonal antibodies [71,77], SFVmac Gag and Pol polyclonal rabbit antisera [78] or APOBEC3G monoclonal antibodies (Santa Cruz) as described previously [46,79,80].

## Competing interests

The authors declare that they have no competing interests.

## Authors' contributions

KG, TW, JP, and AM performed the experiments. AR and CS designed the study. KG, AR, and CS wrote the manuscript. All authors read and approved the final manuscript.

## Supplementary Material

Additional file 1 **Supplementary figures**. Figure S1 – Oligonucleotide primer sequences. Figure S2 – Point mutations found after FV vector transfer. The vectors were produced in the absence or presence (mutations in bold face) of Bet protein. The two A to G transitions are in italics. Figure S3 – Level of human APOBEC3F (A3F) mRNA expression in 293T cells in relation to PBMCs. Figure S4 – One deletion detected among over 265,000 nucleotides analysed. Figure S5 – Number of colonies resistant to Hygro and/or Neo upon transfer of MLV vectors. Figure S6 – Number of colonies resistant to Hygro and/or Neo upon transfer of PFV vectors. Click here for file
