# Supplementary material for: Accuracy estimation of foamy virus genome copying
Source: Retrovirology. 2009 Apr 6;6:32. doi: 10.1186/1742-4690-6-32 (PMC2678077; doi:10.1186/1742-4690-6-32)
Supplement: Additional file 1 — Supplementary figures. Figure S1 – Oligonucleotide primer sequences. Figure S2 – Point mutations found after FV vector transfer. The vectors were produced in the absence or presence (mutations in bold face) of Bet protein. The two A to G transitions are in italics. Figure S3 – Level of human APOBEC3F (A3F) mRNA expression in 293T cells in relation to PBMCs. Figure S4 – One deletion detected among over 265,000 nucleotides analysed. Figure S5 – Number of colonies resistant to Hygro and/or Neo upon transfer of MLV vectors. Figure S6 – Number of colonies resistant to Hygro and/or Neo upon transfer of PFV vectors. [file 1742-4690-6-32-S1.ppt]

## Slide 1
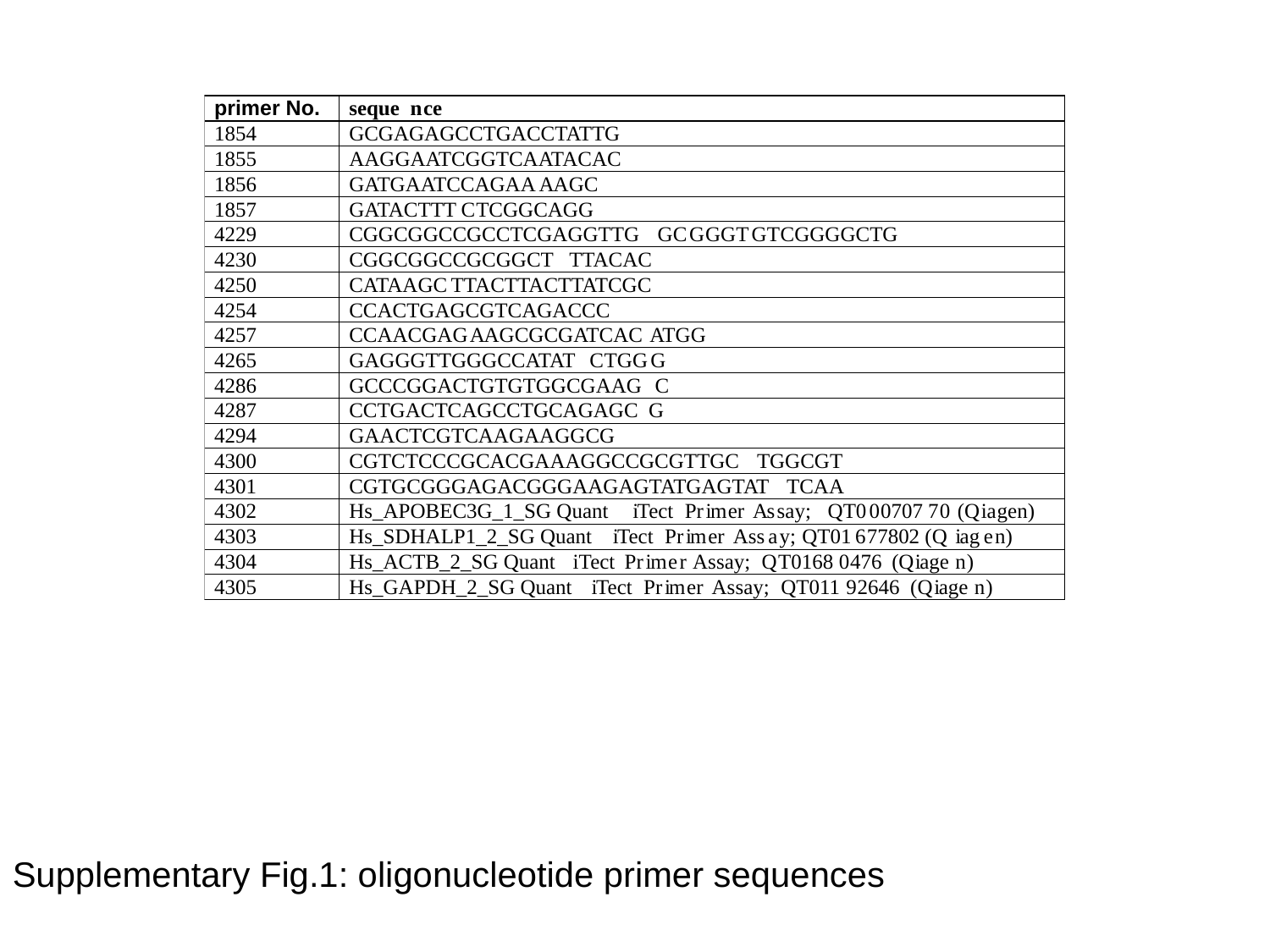

Supplementary Fig.1: oligonucleotide primer sequences

## Slide 2
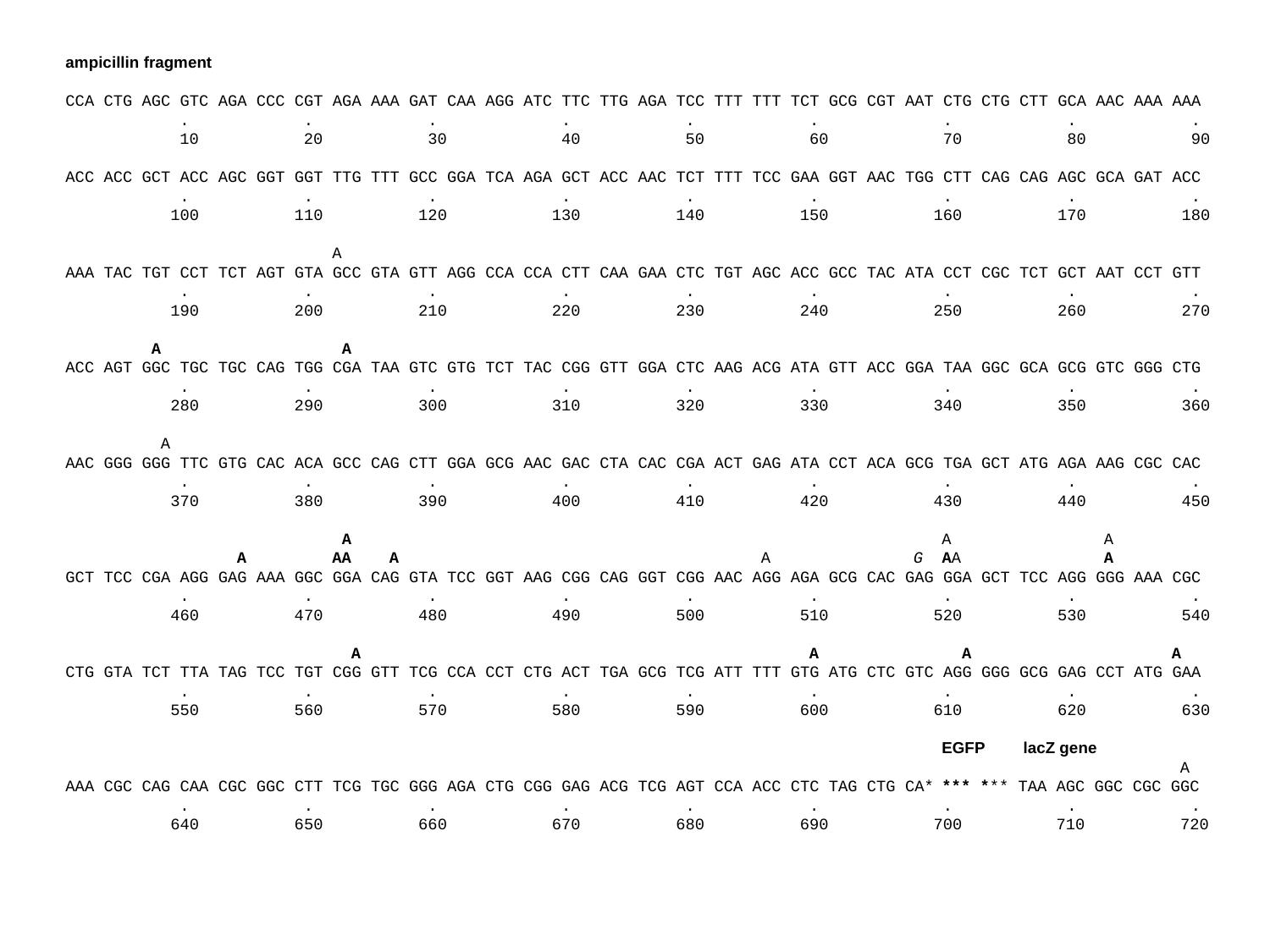

ampicillin fragment
CCA CTG AGC GTC AGA CCC CGT AGA AAA GAT CAA AGG ATC TTC TTG AGA TCC TTT TTT TCT GCG CGT AAT CTG CTG CTT GCA AAC AAA AAA
 . . . . . . . . .
 10 20 30 40 50 60 70 80 90
ACC ACC GCT ACC AGC GGT GGT TTG TTT GCC GGA TCA AGA GCT ACC AAC TCT TTT TCC GAA GGT AAC TGG CTT CAG CAG AGC GCA GAT ACC
 . . . . . . . . .
 100 110 120 130 140 150 160 170 180
 A
AAA TAC TGT CCT TCT AGT GTA GCC GTA GTT AGG CCA CCA CTT CAA GAA CTC TGT AGC ACC GCC TAC ATA CCT CGC TCT GCT AAT CCT GTT
 . . . . . . . . .
 190 200 210 220 230 240 250 260 270
 A A
ACC AGT GGC TGC TGC CAG TGG CGA TAA GTC GTG TCT TAC CGG GTT GGA CTC AAG ACG ATA GTT ACC GGA TAA GGC GCA GCG GTC GGG CTG
 . . . . . . . . .
 280 290 300 310 320 330 340 350 360
 A
AAC GGG GGG TTC GTG CAC ACA GCC CAG CTT GGA GCG AAC GAC CTA CAC CGA ACT GAG ATA CCT ACA GCG TGA GCT ATG AGA AAG CGC CAC
 . . . . . . . . .
 370 380 390 400 410 420 430 440 450
 A A A
 A AA A A G AA A
GCT TCC CGA AGG GAG AAA GGC GGA CAG GTA TCC GGT AAG CGG CAG GGT CGG AAC AGG AGA GCG CAC GAG GGA GCT TCC AGG GGG AAA CGC
 . . . . . . . . .
 460 470 480 490 500 510 520 530 540
 A A A A
CTG GTA TCT TTA TAG TCC TGT CGG GTT TCG CCA CCT CTG ACT TGA GCG TCG ATT TTT GTG ATG CTC GTC AGG GGG GCG GAG CCT ATG GAA
 . . . . . . . . .
 550 560 570 580 590 600 610 620 630
 EGFP lacZ gene
 A
AAA CGC CAG CAA CGC GGC CTT TCG TGC GGG AGA CTG CGG GAG ACG TCG AGT CCA ACC CTC TAG CTG CA* *** *** TAA AGC GGC CGC GGC
 . . . . . . . . .
 640 650 660 670 680 690 700 710 720

## Slide 3
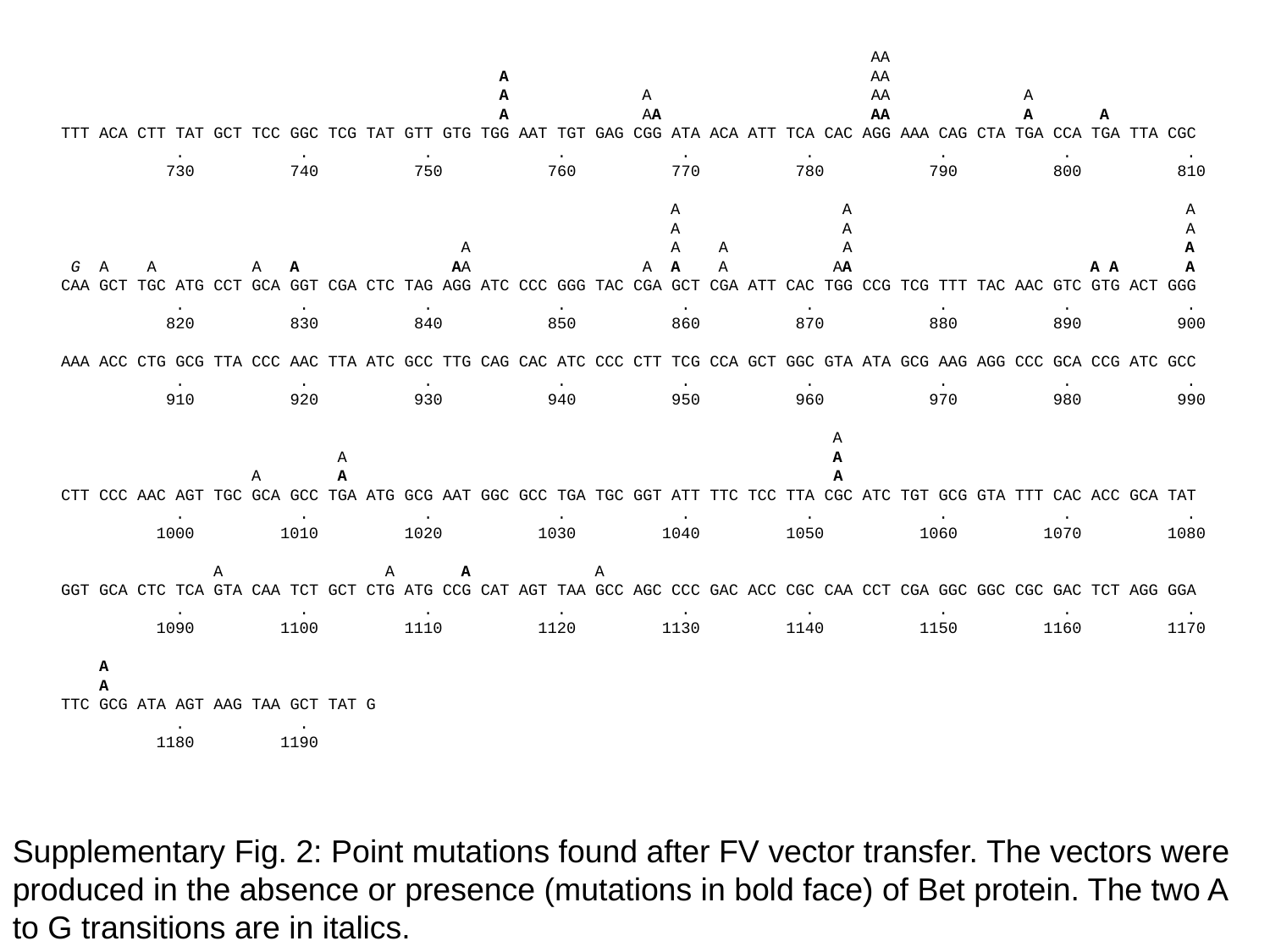

AA
 A AA
 A A AA A
 A AA AA A A
TTT ACA CTT TAT GCT TCC GGC TCG TAT GTT GTG TGG AAT TGT GAG CGG ATA ACA ATT TCA CAC AGG AAA CAG CTA TGA CCA TGA TTA CGC
 . . . . . . . . .
 730 740 750 760 770 780 790 800 810
 A A A
 A A A
 A A A A A
 G A A A A AA A A A AA A A A
CAA GCT TGC ATG CCT GCA GGT CGA CTC TAG AGG ATC CCC GGG TAC CGA GCT CGA ATT CAC TGG CCG TCG TTT TAC AAC GTC GTG ACT GGG
 . . . . . . . . .
 820 830 840 850 860 870 880 890 900
AAA ACC CTG GCG TTA CCC AAC TTA ATC GCC TTG CAG CAC ATC CCC CTT TCG CCA GCT GGC GTA ATA GCG AAG AGG CCC GCA CCG ATC GCC
 . . . . . . . . .
 910 920 930 940 950 960 970 980 990
 A
 A A
 A A A
CTT CCC AAC AGT TGC GCA GCC TGA ATG GCG AAT GGC GCC TGA TGC GGT ATT TTC TCC TTA CGC ATC TGT GCG GTA TTT CAC ACC GCA TAT
 . . . . . . . . .
 1000 1010 1020 1030 1040 1050 1060 1070 1080
 A A A A
GGT GCA CTC TCA GTA CAA TCT GCT CTG ATG CCG CAT AGT TAA GCC AGC CCC GAC ACC CGC CAA CCT CGA GGC GGC CGC GAC TCT AGG GGA
 . . . . . . . . .
 1090 1100 1110 1120 1130 1140 1150 1160 1170
 A
 A
TTC GCG ATA AGT AAG TAA GCT TAT G
 . .
 1180 1190
Supplementary Fig. 2: Point mutations found after FV vector transfer. The vectors were produced in the absence or presence (mutations in bold face) of Bet protein. The two A to G transitions are in italics.

## Slide 4
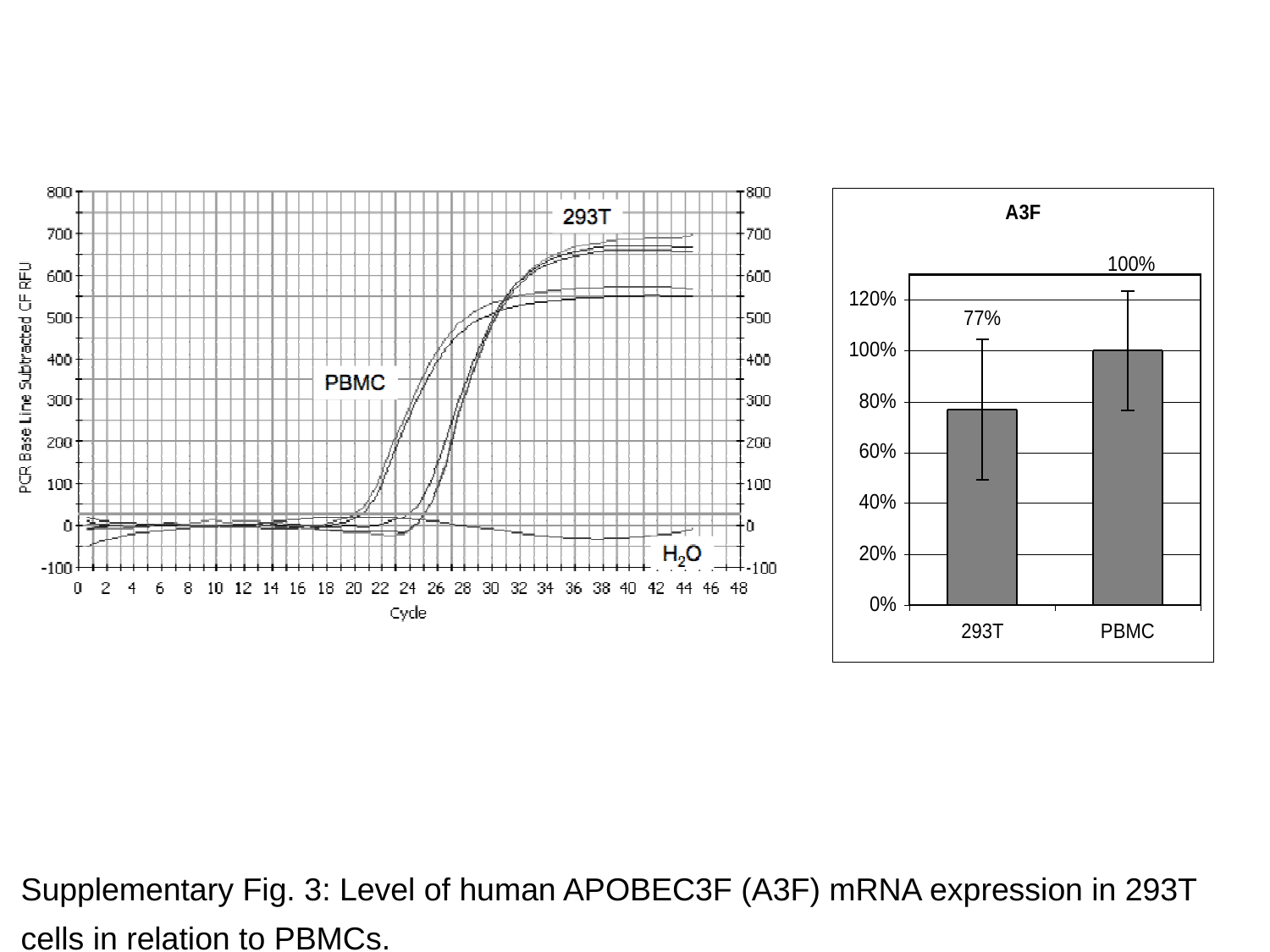

Supplementary Fig. 3: Level of human APOBEC3F (A3F) mRNA expression in 293T cells in relation to PBMCs.

## Slide 5
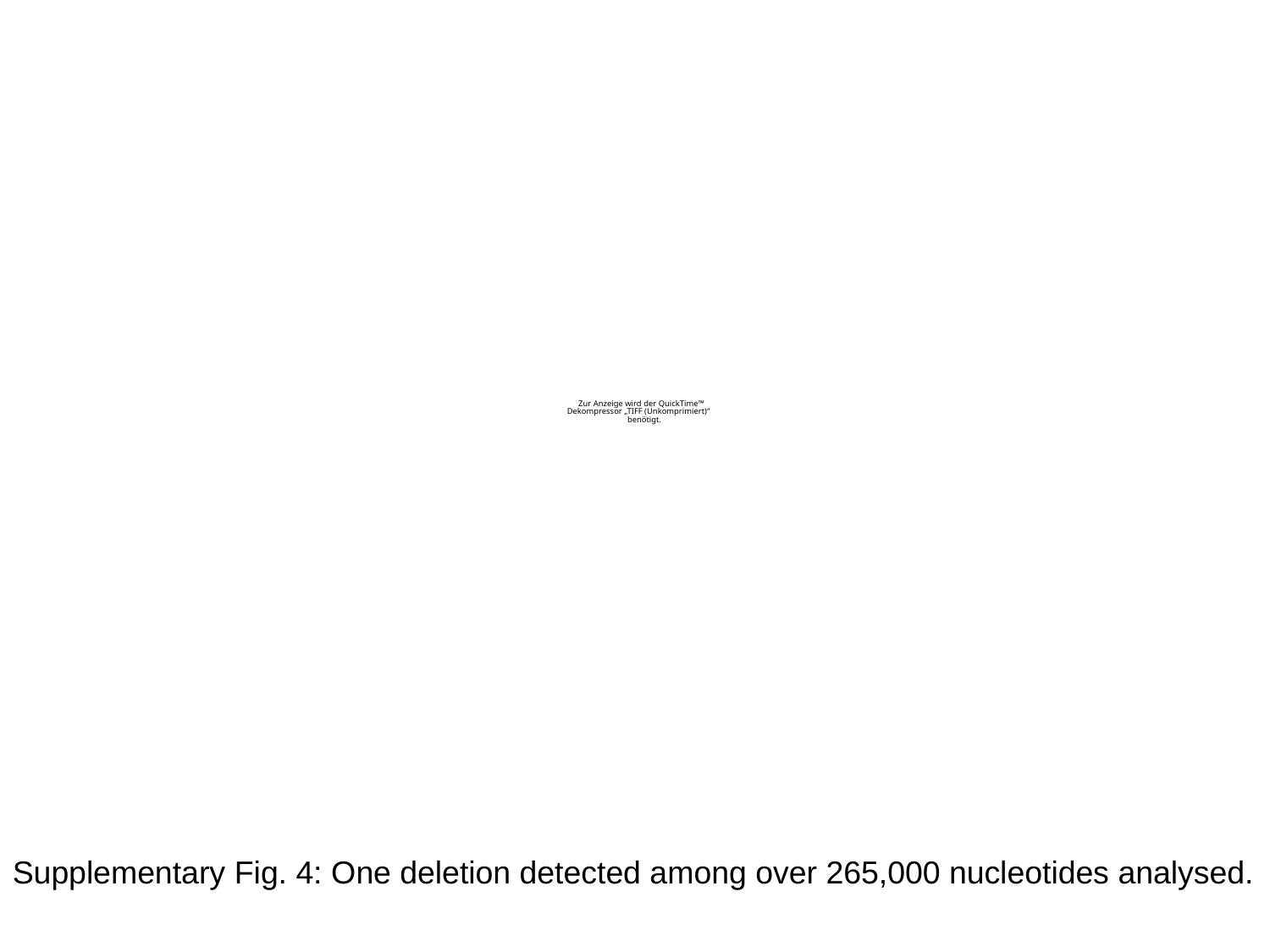

Supplementary Fig. 4: One deletion detected among over 265,000 nucleotides analysed.

## Slide 6
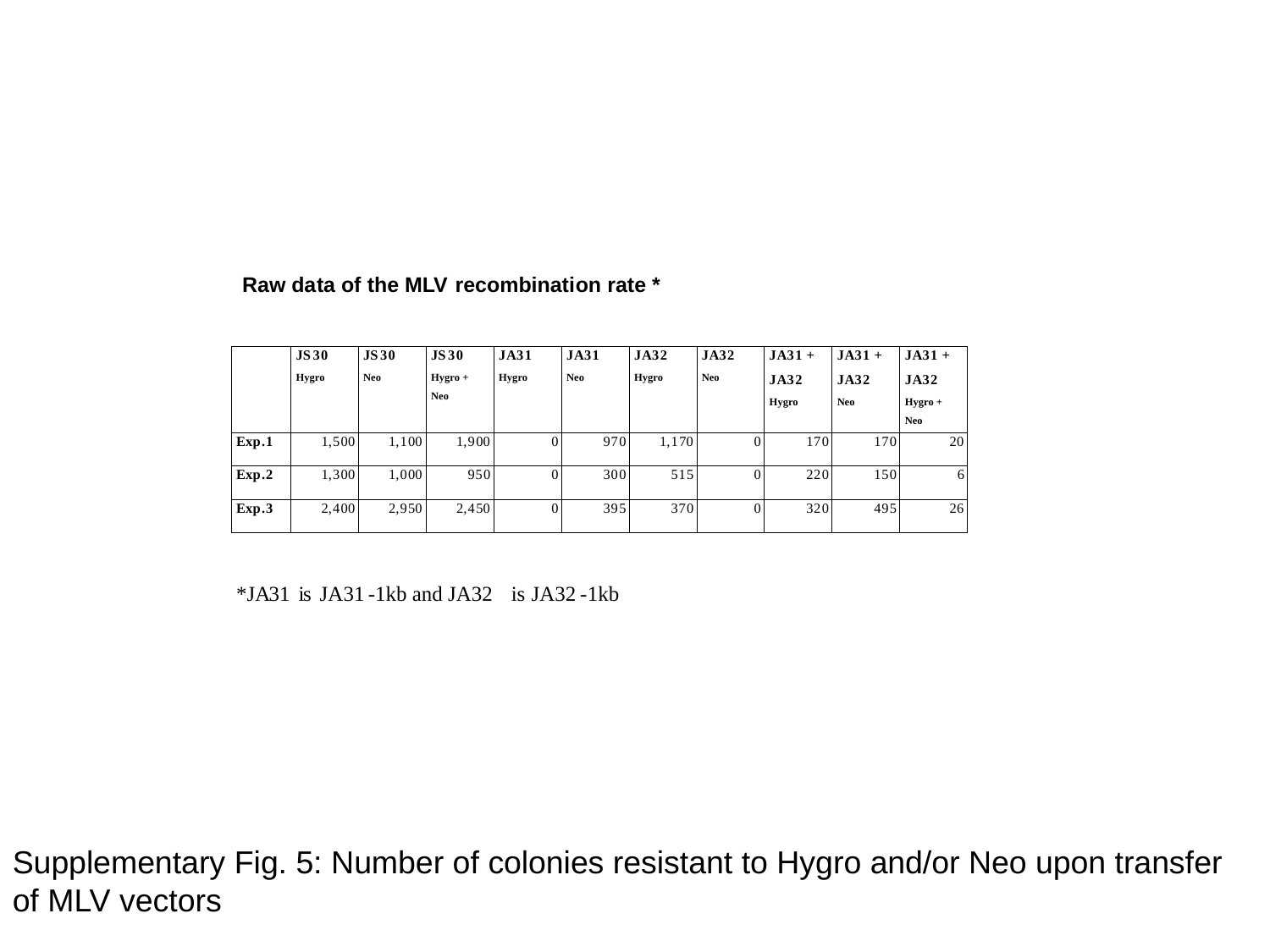

Supplementary Fig. 5: Number of colonies resistant to Hygro and/or Neo upon transfer of MLV vectors

## Slide 7
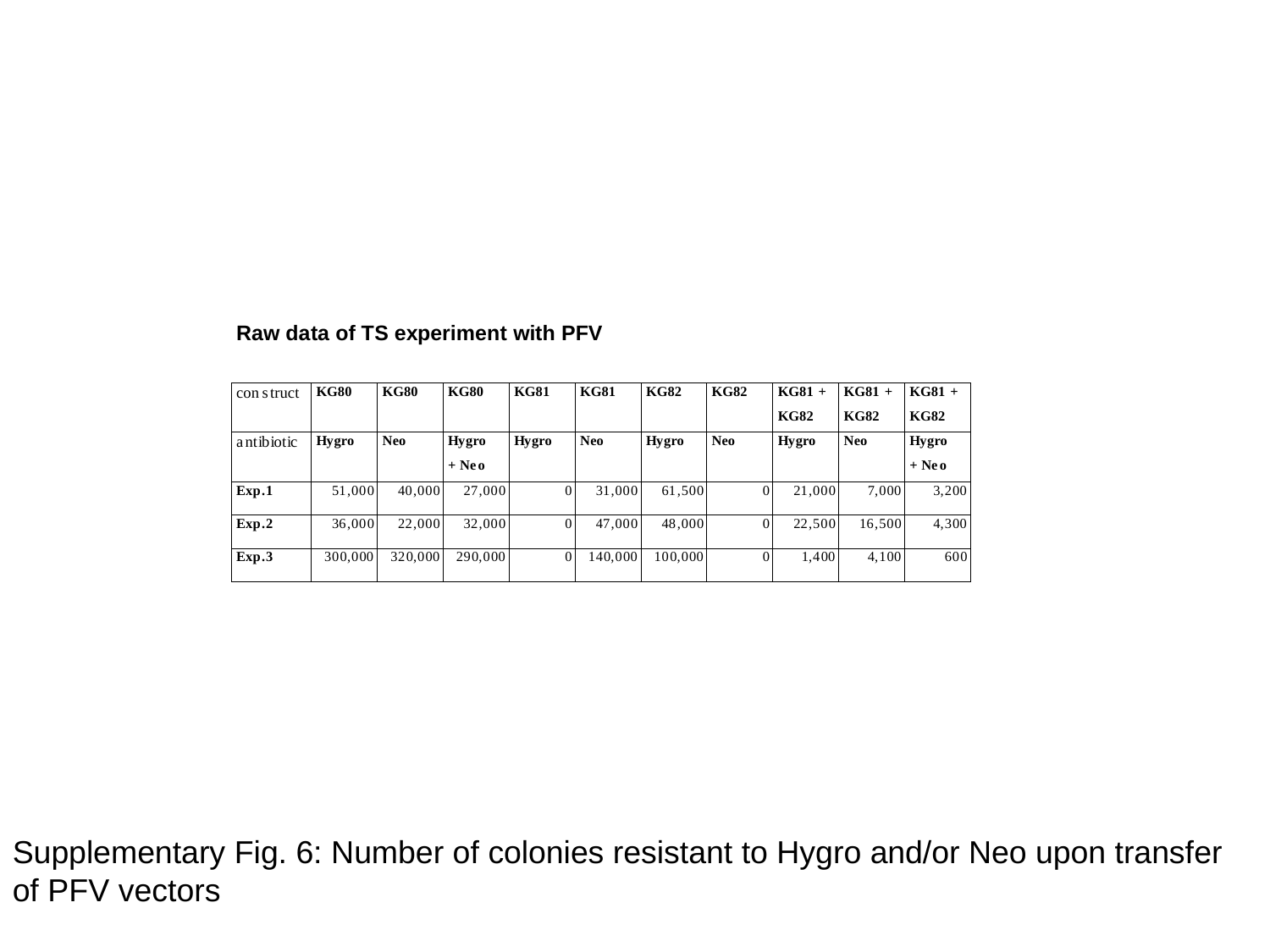

Supplementary Fig. 6: Number of colonies resistant to Hygro and/or Neo upon transfer of PFV vectors
